# Supplementary material for: Unveiling the pentagonal nature of perfectly aligned single-and double-strand Si nano-ribbons on Ag(110)
Source: Nat Commun. 2016 Oct 6;7:13076. doi: 10.1038/ncomms13076 (PMC5059744; doi:10.1038/ncomms13076)
Supplement: Supplementary Information — Supplementary Figures 1-8 and Supplementary Tables 1-2 [file ncomms13076-s1.pdf]

Supplementary Information

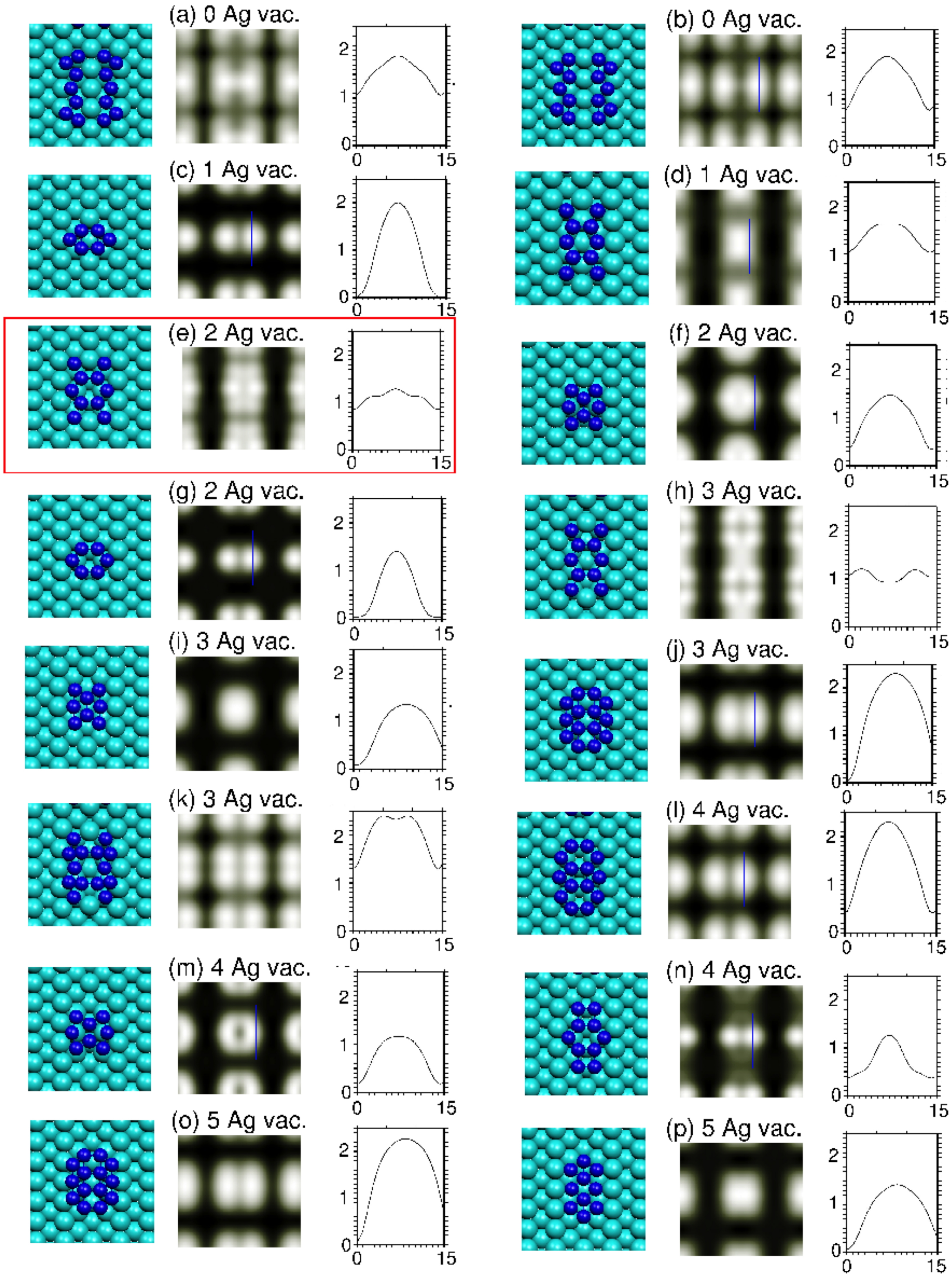

Supplementary Figure 1. Trial nano-dot models: Summary of the most relevant nano-dot trial structures studied in this work employing a reduced (4×5) or (4×6) supercell. Optimized geometries (top view), STM simulations and line profiles along the vertical blue lines shown in the maps for all nano-dot models tested. The models are organized from (**a-p**) with increasing number of silver vacancies, ranging from zero up to five. The structure that best matches the experimental image and line profile given in main text Fig. 2(a) is clearly case (**d**), involving two Ag vacancies and ten Si atoms. The simulated image correctly captures the two large bumps at the center and the dimmer maxima (elbows) above and below them. Furthermore, the associated profile is the only one that resolves the three maxima with just a few tenths of Angstrom difference between the center and the satellite ones, in perfect agreement with the experiment.

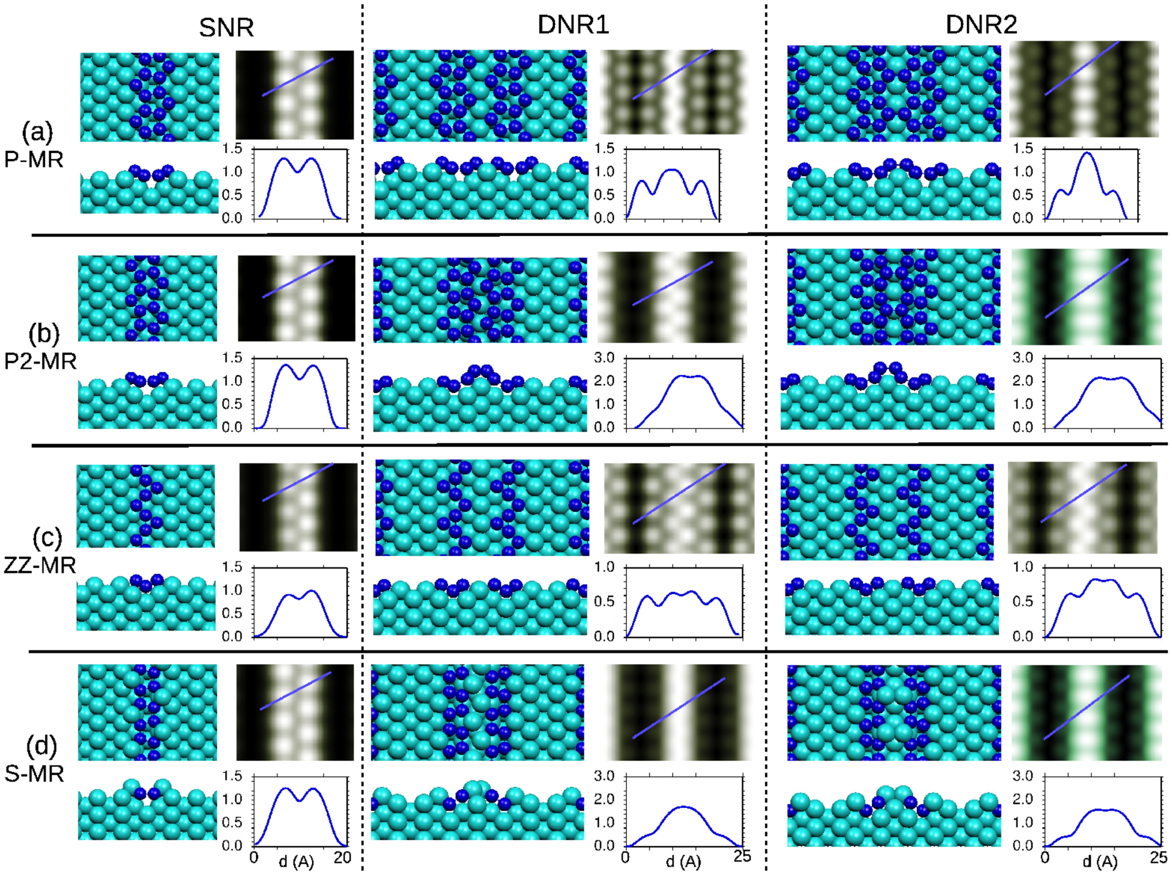

Supplementary Figure 2. Trial MR-NR models: Optimized geometries, STM topographic maps and line profiles for the most relevant MR-based NR structures considered in this work (we omit the tens of models tested based on an unreconstructed Ag(110) surface since they systematically relaxed towards geometries incompatible with the experimental STM images). Left panels correspond to the low coverage SNRs, and center and right columns to the high coverage DNR arrangements following a *–LL–RR–LL–* (DNR1) and a *–LR–RL–LR–* (DNR2) sequence among the enantiomers, respectively. (**a**) P-MR model already shown in Figure 3 in the main text together with the alternative DNR2 arrangement whereby the Si_{ad} become aligned between the adjacent rings and dimerize on top of the short bridge sites leading to a broad ribbon made up of elongated octagons at the center decorated by pentagons at the edges. (**b**) P2-MR model similar to the P-MR one but with the Si_{ad} leaning towards top sites. (**c**) Zig-zag missing row model (ZZ-MR) proposed in Ref. [22]. The motif in this pattern consists of only two Si_s atoms residing in the MR troughs, each of them bonded to another two Si_{ad}s which lean towards the Ag atoms at the top row and protrude out of the surface. (**d**) A substitutional model (S-MR) where the extracted silver atoms attach to the Si_s located in the MR troughs (i.e. equivalent to the P-MR but with the Si_{ad} replaced by Ag_{ad}). Such model would be consistent with a site exchange mechanism between two Si and one silver top row atom without the need for diffusion of the latter across long distances over the surface. All SNRs models yield STM images highly reminiscent of the experimental one (main text Fig. 1(c)), with the only exception of the ZZ-MR structure, which shows an asymmetry in the protrusions due to the lack of a glide plane. However, in both DNR arrangements for the P2-MR and S-MR models as well as in the P-MR DNR2 structure, the adatoms (Si_{ad} or Ag_{ad}) are raised above the silver top row by large distances in order to establish bonds among the adjacent NRs and the corresponding STM images deviate substantially from the high coverage experimental one (main text Fig. 1(e)) as their aspect is now dominated by these high lying Si/Ag atoms. Therefore, these structures may be discarded based on their STM topography. The ZZ-MR DNRs, on the other hand, present a nice correspondence with main text Fig. 1(e), specially the DNR2 arrangement which exhibits a glide plane symmetry. Anyhow, this model may be ruled out as well based on energetic arguments (see Supplementary Table 2 and SupplementaryFig. 4).

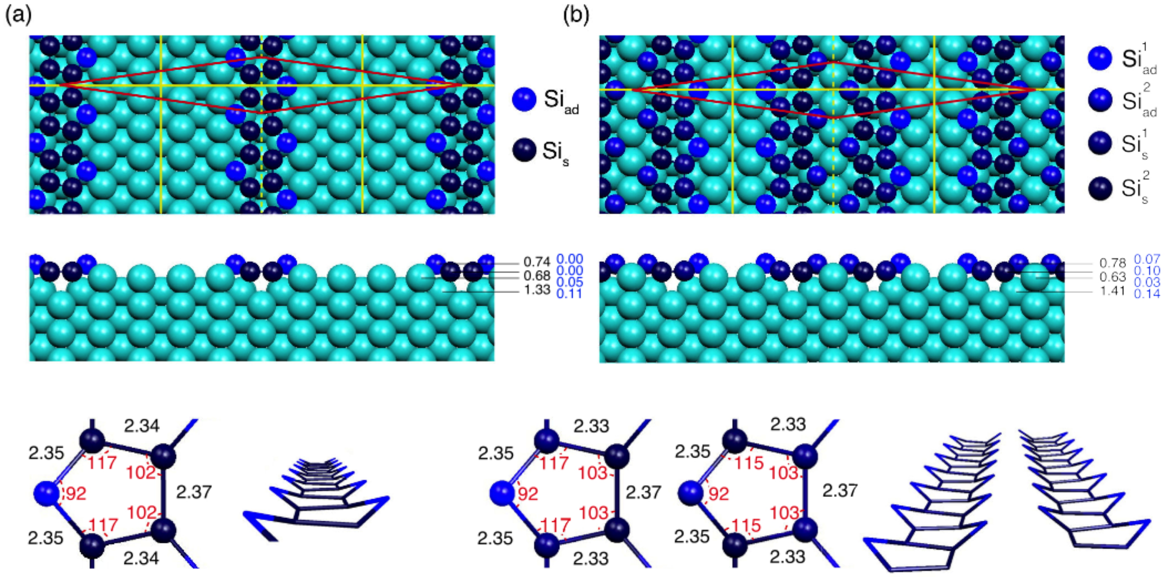

Supplementary Figure 3. Geometry of the P-MR model: P-MR/Ag(110) optimized structures for the Si SNRs (**a**) and the DNRs (**b**). The Si atoms have been colored according to the symmetry inequivalent group they belong (see legends). Top panels: top views with the c(10 × 2) supercell indicated by the dark rhombus while green thick and dashed lines correspond to mirror and glide symmetry planes, respectively. The overall symmetry for both models is cmm. Middle panels: side views with the normal averaged distances between the Si_{ad}, Si_s and the two first silver layers indicated by black numbers, and the buckling within each group of atoms given by blue numbers (all distances in Å). Bottom panels: Zoom in of the pentagonal rings including the Si-Si bond distances (in Å) and bond angles (in red), and perspective views of the 1D pentagonal structures. In Supplementary Table 1 we additionally provide the relative z-coordinate of the Si atoms and their nearest neighbor distances to the metal atoms. In the DNR phase the loss of the local glide plane within each NR makes the two Si_{ad} at each side of the pentagonal chains inequivalent, with the outer ones (Si^1_{ad}) lying 0.06 Å above the inner ones (Si^2_{ad}) while their lateral distance to the top row bridge site is 0.1 Å smaller for the formers. Both trends may be explained from the symmetry constrain on the top row silver atoms along the glide plane, as they cannot shift laterally, as well as by certain Ag-mediated repulsion between the Si^2_{ad} in adjacent NRs (now each top silver atom makes two bonds with the Si^2_{ad}) that shifts the pentagonal rings away from each other by 0.2 Å. Concerning the substrate MR reconstruction there is a 0.1 Å lateral shift of the top row atoms away from the troughs in order to better accommodate the Si NRs. Finally we recall that the fact that the four Si_{ad} in the DNR are not colinear is a key factor in determining the aspect (arrangement of the protrusions) in the STM images.

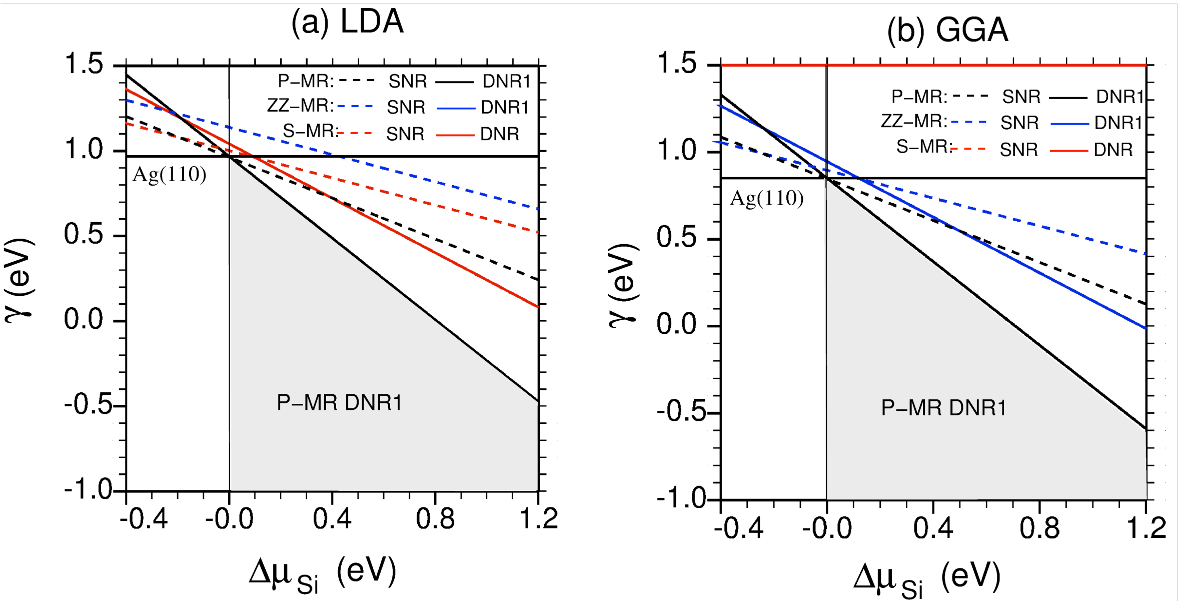

Supplementary Figure 4. Stability of the NR trial models: Phase diagram for the different Si-NR-Ag(110) models studied in this work under the (**a**) LDA and (**b**) GGA. Formation energies, normalized to the Ag(110)-(1 × 1) unit cell, are plotted as a function of the Si chemical potential μ_{Si} according to eq. (3). Note that among those structures with the same number of Si and Ag atoms, we only include the one with largest adsorption energy per Si atom (see Supplementary Table 2), since those omitted run parallel in the plot but shifted upwards. The origin for μ_{Si} has been placed at the first crossing with the formation energy of the clean Ag(110) surface (dark horizontal line) –see Methods for further details. The shaded region indicates the most stable phase for $\Delta\mu_{Si} > 0$, which under both XC functionals corresponds to the P-MR DNR structure. The ZZ-MR and S-MR models, on the other hand, may be ruled out throughout the entire $\Delta\mu_{Si}$ range. Note that the P-MR SNRs start to become more stable for $\mu_{Si} < 0$ in qualitative agreement with the experimental observation, as SNRs are typically formed at small Si coverages, while the DNR phase tends to cover the entire surface as the coverage is increased.

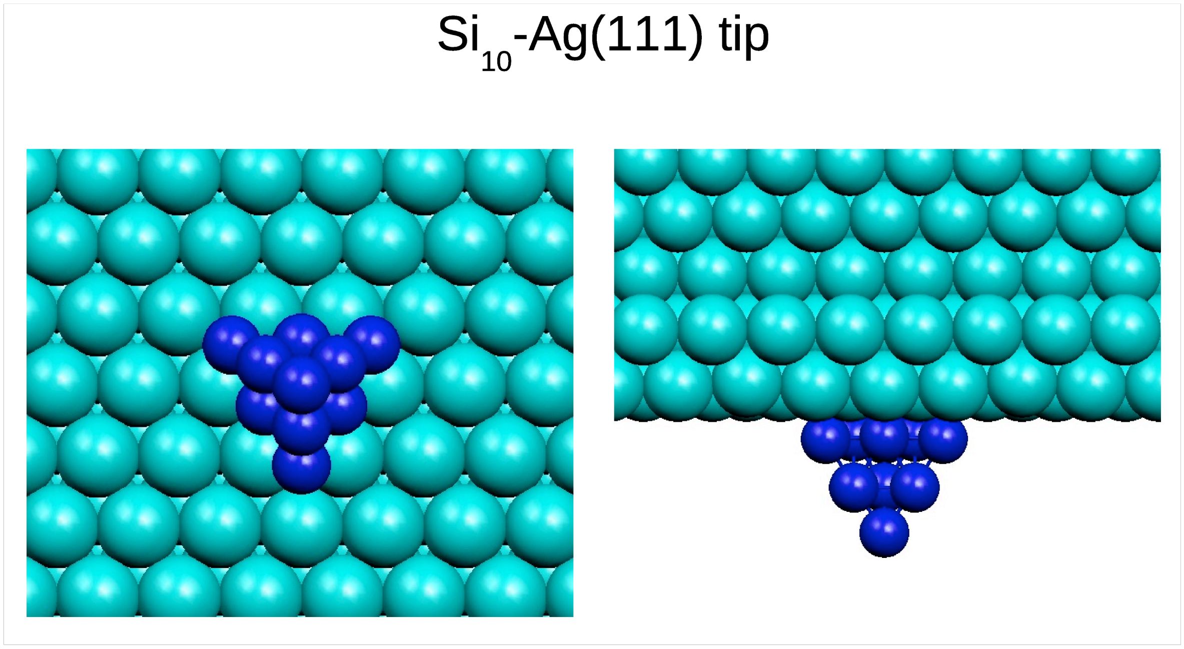

Supplementary Figure 5. Si_{10}-Ag(111) tip: Bottom and side views of the Ag(111) tip terminated in a 10 Si atom pyramid employed for all STM simulations.

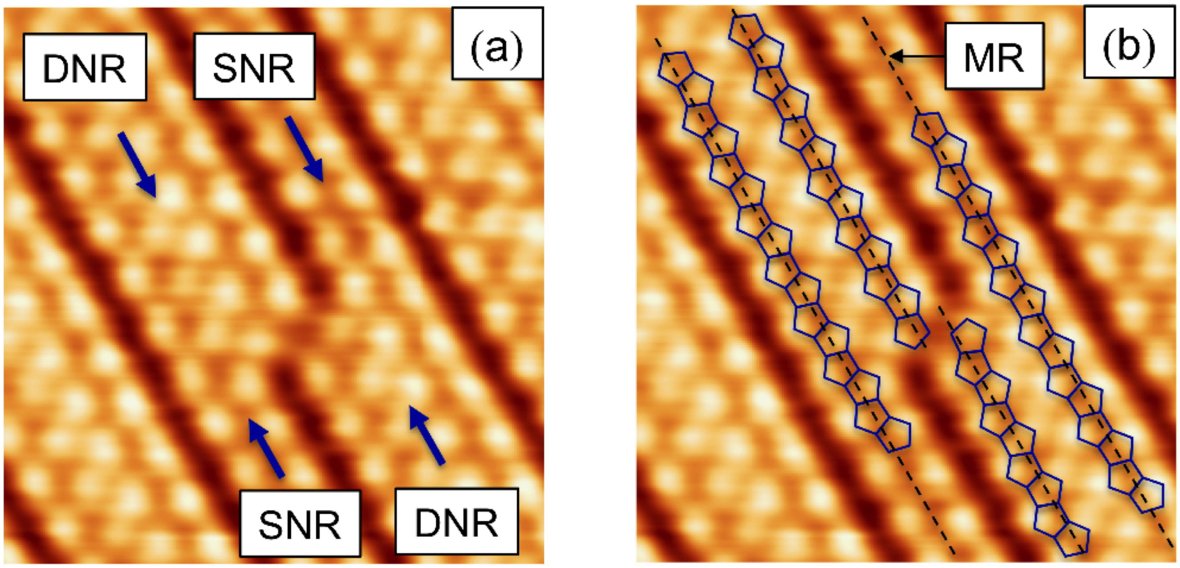

Supplementary Figure 6. **Dislocation defects between NRs**. (**a**) Experimental STM image showing a dislocation within the array of DNRs. The image size is 6.2 × 6.2 nm^2. The sample bias voltage is 0.9 V and the tunnel current 0.8 nA. The aspect of the dislocation is very similar to that reported in previous STM works [22]. (**b**) Same as (a) after superimposing the Si pentagons (blue) and the MRs (dashed solid lines). The upper and lower truncated MRs at the center are shifted from each other by one Ag lattice parameter, leading to a DNR-SNR arrangement at the top of the image and a SNR-DNR at the bottom. Indeed, this type of dislocations are typically found when a SNR appears between two DNRs, in which case the (5x2) periodicity is truncated. Since from the STM images there does not seem to exist a point defect at the dislocation which could drive the generation of the shifted MRs, we speculate that each of them is created far away and upon further growth the MRs meet thus forming the dislocation.

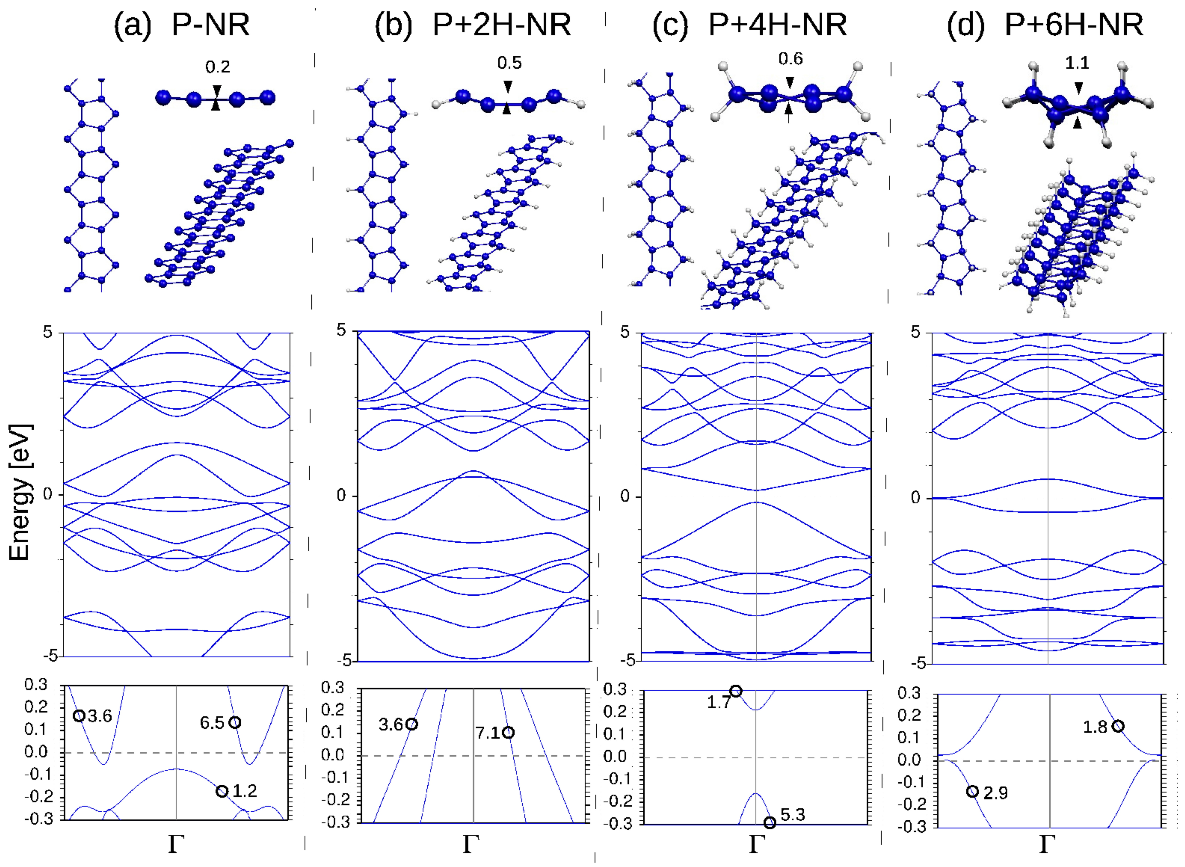

Supplementary Figure 7. **Energetic comparison between hexagonal and pentagonal MR structures stabilized on Ag(110)**: In order to address the energetics between the Si pentagonal (P) and nano-dot quasi-hexagonal (H) motifs, several models with different number of Si atoms located in extended troughs (MRs) and under both P and H configurations have been optimized: (**a**) a 10 Si atom cluster, (**b**) a 14 Si atom cluster, (**c**) a 20 Si atom cluster and, (**d**) the 1D version of (a) with 6 Si atoms per (4 x 2) cell. Within each panel, left and right columns provide top views of the optimized structures under the H and P configurations. The arrows in the middle point to the most stable structure, with the total energy difference given above in eV/Si. Below the arrows the unit cell employed for each pair of calculations is indicated. In (**a**) the H structure is more stable than the P one, since the lower Si pentagon cannot be closed –the number of Si atoms required to obtain closed pentagonal clusters is $N_c = 5 + 3m$. The situation is reversed in (**b**) and (**c**), where the P clusters are closed (m = 3 and 5, respectively) and become much more stable than the H structure as the number of Si atoms is increased. Note that all P clusters converge to a similar edge structure whereby the end pentagons rotate so that the dangling Si_s atoms end up at high-symmetry sites in order to reinforce their bonds to the metal substrate: one of them close to a four-fold site and the other one at a second layer bridge site leaning towards a top row atom. Finally, in (**d**) the P strands are again much more stable than the H counterparts. The comparison therefore reveals that pentagons are more favorable on the Ag(110) surface than a mixture of hexagonal and square motifs. The calculations additionally reveal that H clusters or strands remain metastable in the MR troughs, suggesting that the hexagonal-to-pentagonal transition is not spontaneous upon removal of top row silver atoms but most probably involve complex (multiple) exchange processes among the Si and Ag atoms.

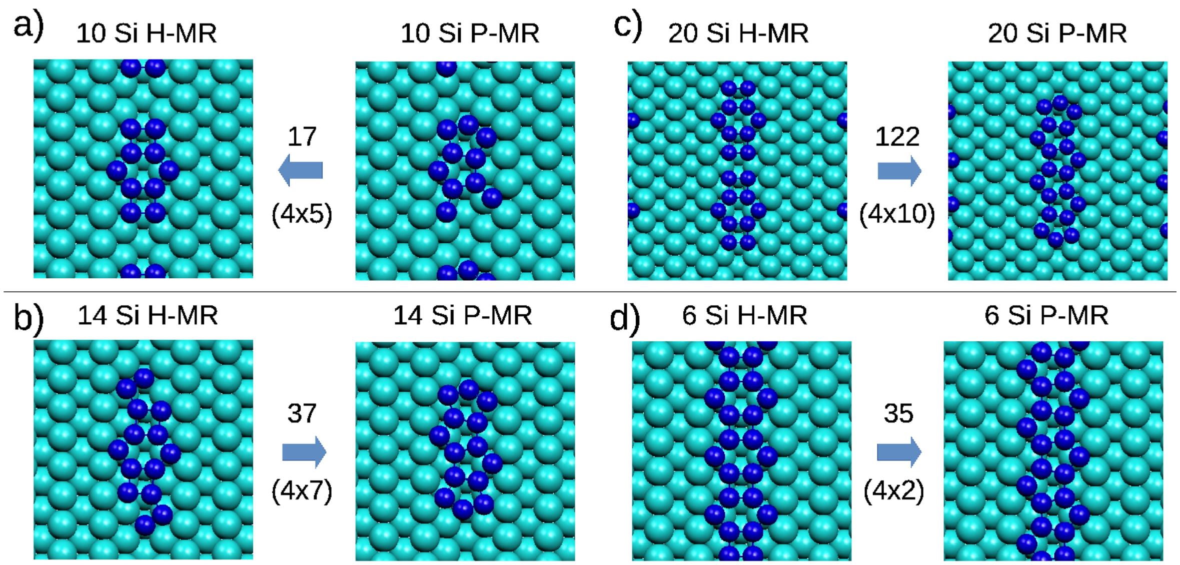

Supplementary Figure 8. **Structure and electronic properties of free-standing pristine and hydrogenated Si pentagonal NRs**: (**a**) A pristine Si pentagonal strand, (**b**) same as (a) after saturating with one hydrogen the edge atoms (Si_{ad}) at each side of the NR, (**c**) same as (b) but with two H atoms per Si_{ad} instead, (**d**) same as (c) but also saturating two of the inner Si_s atoms with two additional H atoms. Top panels: top, side and perspective views of the free-standing penta-silicene nanoribbons (P-NRs). The overall buckling of the Si pentagon is indicated in Å in the side views. The pentagons are almost flat in the free-standing case, while the buckling increases as more H is added to the NRs –notice that the pentagonal structure of the P+2H structure in highly reminiscent of the P-MR on Ag(110) model, while in the P+4H and P+6H cases there is an additional buckling among the inner Si_s. The cohesive energy of the pristine pentagonal strand is 4.9 eV/Si which amounts to almost 80% of the computed adsorption energy for the P-MR structure on Ag(110) (6.4 eV/Si –see Supplementary Table 2) implying that, energetically, the main stabilization mechanism for this phase is the Si-Si pentagonal bonding rather than the interaction with the Ag(110) substrate. In the P+nH-NR structures, the computed NR-H interaction energy, according to eq. (4), was found to be 1.4, 2.9 and 4.1 eV/Si for (b), (c) and (d), respectively –that is, the strength of the Si-H bond is roughly 0.7 eV in all cases. Notably, for the P+4H and P+6H structures these interaction energies are much larger than that for the NR-Ag(110) system (1.6 eV). Middle panels: the corresponding band structures. Bottom panels: zoom into the band structures in the vicinity of the Fermi level. Overall, the effect of adding H to the NR is to shift away the Si-bands away from the Fermi level. All structures result metallic except the P+4H which shows a direct gap at $\bar{\Gamma}$ of 0.38 eV. In the free-standing case (a) two deep electron pockets appear close the the 1D BZ edge, while the P+6H NR presents two shallow hole pockets at the BZ boundary.

In the P+2H NR structure (b) four almost linear bands cross the Fermi level. In the lower panels, the Fermi velocities, $\mathbf{v}_F = \frac{1}{\hbar}\frac{\partial E(\mathbf{k})}{\partial \mathbf{k}}$, evaluated at the linear regions of the band structures (marked by circles) are given in units of $x10^5$ m s^{-1}.

	atom	z(Å)	$d_{\mathrm{Si-Ag}}$(Å)
SNR	$\mathrm{Si_{ad}}$	1.42	2.56 (x2), 2.80
	$\mathrm{Si_s}$	0.68	2.58, 2.74
DNR	$\mathrm{Si_{ad}^1}$	1.44	2.55 (x2), 2.88
	$\mathrm{Si_{ad}^2}$	1.38	2.56 (x2), 2.78
	$\mathrm{Si_s^1}$	0.68	2.58, 2.73
	$\mathrm{Si_s^2}$	0.58	2.58, 2.76

Supplementary Table 1. Details of the P-MR/Ag(110) geometry: Relative vertical distances (z) with respect to the average top-most Ag layer and bond distances to the first silver nearest neighbor ($d_{\mathrm{Si-Ag}}$) for each of the symmetry inequivalent atoms in the SNR and DNR structures –see Supplementary Figure 3 for further details.

Model	SNR			DNR1 (*–LL – RR – LL*)			DNR2 (*–LR – RL – LR*)		
	$N_{\mathrm{Si/Ag}}$	$E_{\mathrm{ads}}^{\mathrm{LDA}}$	$E_{\mathrm{ads}}^{\mathrm{GGA}}$	$N_{\mathrm{Si/Ag}}$	$E_{\mathrm{ads}}^{\mathrm{LDA}}$	$E_{\mathrm{ads}}^{\mathrm{GGA}}$	$N_{\mathrm{Si/Ag}}$	$E_{\mathrm{ads}}^{\mathrm{LDA}}$	$E_{\mathrm{ads}}^{\mathrm{GGA}}$
P-MR	6/88	**6.44**	**5.70**	12/86	**6.43**	**5.70**	12/86	6.40	5.67
P2-MR	6/88	6.24	5.56	12/86	6.23	5.68	12/86	6.41	5.49
ZZ-MR	4/88	6.36	5.59	8/86	6.35	5.58	8/86	6.34	5.58

Supplementary Table 2. Si adsorption energies: Adsorption energies per Si atom, in eV, obtained both under LDA and GGA according to eq.(1) for the different MR models considered (except the S-MR). They range between 6.2–6.4 for LDA and 5.5–5.7 eV for GGA. The large 0.7 eV difference between the two functionals is caused by their well known over- and under-binding nature, respectively. Nevertheless, both functionals point to the P-MR SNR and DNR as the most stable ones (marked in bold face in the table) the two attaining very similar values.
